# An Illegal Practitioner Sent to Jail

**Published:** 1895-11

**Authors:** 


### An Illegal Practitioner Sent to Jail.

In New York City, the lawyers employed by the County Medical Society have secured the conviction of a "Doctor" Ruffo for practicing medicine without a license and registration. He was sentenced to a three months' term in the penitentiary.

